# The Role of Siblings in Talent Development: Implications for Sport Psychologists and Coaches

**DOI:** 10.3389/fspor.2021.626327

**Published:** 2021-02-16

**Authors:** Robin D. Taylor, Dave Collins, Howie J. Carson

**Affiliations:** ^1^Institute for Coaching and Performance, University of Central Lancashire, Preston, United Kingdom; ^2^Institute for Sport, Physical Education and Health Sciences, Moray House School of Education and Sport, The University of Edinburgh, Edinburgh, United Kingdom; ^3^Grey Matters Performance Ltd., Stratford-Upon-Avon, United Kingdom

**Keywords:** coaching, family, professional practice, biopsychosocial development, psychology, talent development environments, sibling characteristics

## Abstract

Developing talent requires consideration of social networks that can facilitate or inhibit progression. Of fundamental influence in this regard is the family, with recent investigation extending its focus from parents to the role of siblings. As such, the purpose of this *Conceptual Analysis* article is to, firstly, review the characteristics of the sibling relationship that may support or inhibit talent development. Secondly, the analysis then provides empirically derived practical examples to emphasize the holistic and complex role that siblings can play in talent development. Thirdly, strategies are proposed to support practitioners identify specific sibling characteristics, alongside recommendations for how the relationship can be utilized within both the formal and informal environments by coaches and psychologists. Finally, and crucially, important implications of these characteristics are considered to support effective coach and sport psychologist decision making.

## Introduction

Talent development (TD) refers to “a multi-faceted process of optimally nurturing athletes over time within a sport-system” (Cobley et al., 2021, p. 8). As such, and in recognizing that TD is usually a long-term process, TD environments (TDEs) are recommended to focus on introducing and building pertinent skill-sets. Indeed, these skills should enable adaptability across the talent pathway with the aim of achieving high performance outcomes (Martindale and Mortimer, 2011). Specifically, optimizing the impact of formal experiences, such as adult-led coaching sessions, and working through informal environments, such as child-led activity in gardens, should be considered by practitioners to appropriately assist athletes toward reaching their potential in what is typically a non-linear and challenging pathway (Collins and MacNamara, 2018). Reflecting the emphasis of this paper, siblings can be important and unique developmental agents within these contexts when compared to coaches, parents, psychologists, and peers (Collins et al., 2016; Blazo and Smith, 2018). However, there is also the potential for siblings to have no impact, or a negative impact, on development (Weissensteiner et al., 2009; Warmenhoven et al., 2020). Undoubtedly, one argument which is often applied to siblings is the nature vs. nurture debate. This is, of course, of academic interest, although examining intact families with siblings is clearly not informative. The situations encountered are clearly an indivisible combination of nature *and* nurture (cf. Mark et al., 2017). For research purposes, psychologists have focused on a particular type of sibling, namely twins (e.g., Segal, 2012) and ideally, twins separated in early life (Segal et al., 2015). This is not a concern of this paper however, rather its aim is to highlight issues and methods which might be useful for coaches and psychologists when working with sporting siblings.

Surprisingly, despite widespread and longstanding observance of sibling involvement within cases of elite sporting success, how these relationships may best support TD has only recently formed an explicit focus within the coaching literature. Therefore, this paper aims to focus on the sibling relationship by reviewing empirically derived characteristics, its impact on holistic development, and its complexity. Finally, the paper will conclude by discussing how sport psychologists and coaches can identify and positively utilize a sibling relationship for TD across formal and informal environments.

## Reviewing Characteristics of the Sibling Relationship

Several studies have considered the role of siblings across sport participation (i.e., participation, development, and performance), suggesting that siblings can have a meaningful influence on an athlete in both a positive and negative way (Côté, 1999; Davis and Meyer, 2008; Blazo et al., 2014; Trussell, 2014; Hopwood et al., 2015; Allbaugh et al., 2016; Nelson and Strachan, 2017; Osai and Whiteman, 2017; Taylor et al., 2017, 2018, 2021). Reflecting this polar influence, an initial retrospective study by Côté (1999) investigated the historical family dynamics of four 18-year old elite junior level athletes throughout their developmental years. Results revealed positive role modeling behaviors between siblings, that included an influence on the elite-level athlete's decision to specialize within a sport. Significantly, there were also negative emotions reported, such as bitterness and jealousy, from other siblings due to the family shifting attention more toward the talented athlete. Expanding on this, Davis and Meyer (2008) qualitatively examined current adult experiences of 10 elite level sibling-athletes (aged 18–27 years old) through a psychological lens during competition against one another. They identified sibling competitiveness as being different from other opponents. Specifically, this was characterized by previously unidentified processes, again interpreted by the authors as positive (i.e., rivalry, closeness, and respect) by providing emotional support and motivation, as well as negative in the form of gloating.

In broader sport participation studies, Fraser-Thomas et al. (2008) examined the outcomes of dropout vs. prolonged engagement in adolescent competitive sport. These authors identified a differential role of siblings when comparing these outcomes. Those dropping out highlighted high levels of competition, rivalry, and jealousy, while those that maintained engagement experienced generally positive role modeling from their sibling. Furthermore, Weissensteiner et al. (2009) made the case for acknowledging the socio-developmental environment as part of a conceptual model of expertise in cricket. Within this model they highlighted the role of siblings through competition, thereby supporting the development of psychological attributes such as competitiveness, strategizing, coping, and mental toughness. At this point in the literature the sibling relationship in sport can now be seen to be complex in nature.

Next, Blazo et al. (2014) investigated 10 participants (aged 18–32 years old) who had at least one older sibling on an athletic scholarship. Participants themselves either played the same sport as their older sibling, played a different sport, or did not play sport beyond youth recreational leagues. They highlighted the positive impact siblings might have on achievement in sport, suggesting that the relationship had a broader positive family and social influence; as well as helping to develop fondness of another sibling and the development of an identity, whether shared or individual. Similar to previous studies, Blazo et al. (2014) also identified negative connotations such as abandonment and jealousy, which further established the sibling influence as both broad and diverse.

The first quantitative exploration of siblings in sport, conducted by Hopwood et al. (2015), concluded that siblings may play an important role in the development of sporting expertise. Two hundred and twenty-nine athletes (aged 15–35 years old), classified as elite, pre-elite, or non-elite, identified associations between sport expertise, sibling characteristics, and sibling participation in sport and physical activity. Findings suggested that elite athletes were less likely to be first born children, while siblings of elite athletes were more likely to have participated in sport at the pre-elite and elite levels. Similarly to previous qualitative studies, this research revealed the positive older sibling influence on psychological and social factors. Subsequent qualitative studies by Osai and Whiteman (2017) and Nelson and Strachan (2017) explored the potential impact of siblings on TD. Both studies attributed siblings' active engagement to the enhancement of skills and abilities. Nelson and Strachan added that athletes participating in the same sport as their siblings developed a much deeper understanding of each other and their experiences within sport, with the sibling role being potentially both positive (relationship growth and understanding) and negative (sibling competition and emotional response).

Recently, Taylor et al. (2017, 2018, 2021) advanced the study of siblings and TD by tracking athletes across meaningful timeframes and at relevant ages. Initial investigation retrospectively explored the impact of siblings from retired athletes who had competed at the Olympic Games, World Championships or professionally. Data highlighted the perceived importance, and holistic role of siblings during development (Taylor et al., 2017). Consequently, further qualitative study tracked junior athletes (aged 8–16 years) longitudinally during the TD process, combining data from the siblings with parent perceptions to encapsulate the wider family interpretation of the process (Taylor et al., 2018, 2021). All three studies by Taylor and colleagues outlined a number of characteristics perceived to be positive mechanisms for TD, which supported and expanded on the findings from previous studies (see Table 1). Importantly, analysis of the findings illuminated the presence of biopsychosocial interactions resulting from important contextual information when interpreting these data across time and sibling dyads.

**Table 1:** Empirically derived characteristics of the sibling relationship.

**Characteristics**	**Components**	**Examples in action**
Interactional context	Competition Practice Play Recreation	Competing at same level Skill training Sport-focused play at home Involved in different sports
Emotional interpersonal skills	Closeness Comfort Empathy Support	Spending time together First person they go to for help Understanding if something goes wrong Encourage each other
Rivalry	Competitiveness Motivation Success Performance Affective response	Do as well as each other Learning driven by each other Frustrated if sibling won Want to do better than the other Lose temper in defeat
Skill development	Mentoring Co-operation Observation Challenge	Help each other improve through guidance Bounce ideas off each other Watch sibling do a skill Skill challenges against each other
Communication	Instruction Discussion Feedback	Tell sibling how to get better What they are doing, what they need to do Evaluate skills/progress
Conflict	Arguments Frustration Criticism	Disagreement about performance Annoyed they do not agree Tell sibling what they did not do well
Resilience	Ambition Development Test Behavior Mental process	Sibling at the level the other wants to be Harder on each other to help improvement Dealing with failure/loss against sibling Learn to take criticism or let it affect them Develop mental toughness
Identity	Shared Self	Embrace being compared as siblings Develop individual niches in sport
Separation	General Sport-specific Self-initiated Distance	Have different social groups Avoid training together Choosing not to talk about the match Going to different competitions

Finally, a quantitative questionnaire study conducted by Warmenhoven et al. (2020) explored the different types of support and support providers utilized during the development of male cricket players across different levels of skill expertise. Data highlighted that 77% of siblings were important fellow participants in sport. Furthermore, siblings of elite when compared to community cricketers, were more likely to provide access to coaching and technical advice, while also identifying appropriate drills for skill development and supporting the setup of such environments (Warmenhoven et al., 2020). Therefore, based on these latter studies, utilizing, or even developing, characteristics that are positive for TD through the sibling relationship, might be considered as beneficial both within and away from the formal coaching environment (cf. Casey and Goodyear, 2015; Taylor et al., 2018).

To summarize, the literature focusing on siblings and TD is in its infancy, yet some clear considerations are emerging. Specifically, this relationship seems consistently complex and diverse. Research highlights that no individual relationship looks or works in the same manner, nor that this will remain constant across all ages. Furthermore, there is important evidence suggesting that the sibling relationship can support a range of skills to underpin TD. Evidence has predominantly addressed psychological and social influences a sibling can have on TD through formal and informal settings, although with much less attention has been directed toward biological or motoric development. For psychologists and coaches to best contextualize, rationalize, and utilize the relationships in practice, a stronger appreciation of how the psychological, social, and biological disciplines interact, is needed (Bailey et al., 2010).

## Recognizing the Holistic Nature of Sibling Relationships: Optimal Interpretations Through an Interdisciplinary Perspective

As identified, the relationship characteristics (see Table 1) are expressed as a result of interactions from different disciplines that underpin TD; namely, biological, psychological, and social. Indeed, Abbott et al. (2005) identify environments that do not acknowledge or encourage such a multifaceted developmental approach, as risking the quality of an appropriate environments to compliment the complex, dynamic, and non-linear reality of professional sport. Consequently, a deep and broad understanding of these interactions will afford more effective interventions across formal and informal TDEs; as opposed to only focusing on physical or social development in isolation. For example, in the context of a performance review, which is a process most coaches and psychologists will be familiar with, consideration of biopsychosocial interactions might demonstrate: *Reflective discussion between siblings about what happened, why, and how it could be improved (psychological), re-enacting the skill as it would be intended to in the future (biological), followed by reinforcement and support from the sibling to ensure that it takes place (social)*. Accordingly, the review process is not simply a desk-based activity, but a process of thinking, doing, and sharing, which can then be continued post-session between coach/psychologist-athlete and athlete-sibling through monitoring procedures.

Such an example acknowledges how siblings can create contexts that support the biopsychosocial development of an athlete. Empirically, Taylor et al. (2017, 2018, 2021) identified relationship characteristics that exemplify how holistic development can take place. In addition to the work of Taylor and colleagues, Davis and Meyer (2008) suggested that the motivations some siblings gain from being compared can fuel an increase in both physical and mental training workload. Similarly, Weissensteiner et al. (2009) highlighted the relationships impact on the psychological skills of strategizing, coping, and mental toughness. Furthermore, Allbaugh et al. (2016) suggested positive social relationships between siblings are more likely to influence positive behaviors. Finally, Nelson and Strachan (2017) identified that the psychological impact of a competitive relationship can create an emotional response, developing a more meaningful social bond.

These studies have consistently recognized and explained interactions between disciplines, with arguably Taylor et al. providing the only purposeful biopsychosocial lens through which to frame these interactions. Notably, there is increasing recognition within TD of the important development of skills within these disciplines alongside, and underpinning, sport-specific technical and tactical development (Bailey et al., 2010). As such, for research and practice to continue to expand within TD, exploring the impact of significant others (e.g., siblings, psychologists, coaches) on the biopsychosocial athlete development athlete, is crucial.

## Treading Carefully: The Complexity of the Sibling Relationship

As Taylor et al. (2018, 2021) highlight, it is important to be cautious when considering the role of siblings in supporting TD. As such, there is increasing evidence showing the difference in the nature, and importance, of specific characteristics both within, and across, sibling dyads (Fraser-Thomas et al., 2008; Taylor et al., 2018; Warmenhoven et al., 2020). With such variety reinforcing the notion that this relationship requires careful consideration over time (Blazo et al., 2014; Allbaugh et al., 2016; Nelson and Strachan, 2017; Taylor et al., 2017). This view reflects Cruickshank and Collins's (2016) appeal for practitioners to take an “it depends” approach when intervening (e.g., sibling competition or collaboration) in the pursuit of a relevant biopsychosocial development outcome, within a specified context. Within the context of the sibling relationship during TD, it is important to affirm the meaning of such a phrase; with “it” being the impact a sibling can have on TD, and, “depends” representing the need to understand the differences in what characterizes a specific relationship and a consideration intervention timing (e.g., pre-season or mid-season).

For example, when considering the role and impact of rivalry within a sibling relationship, several studies have identified paradoxical considerations. Davis and Meyer (2008) suggested that sibling competition may only benefit some athletes' performances. Taylor et al. (2018) further demonstrated that levels of reported rivalry differed across sibling dyads (e.g., brother-sister dyad reported less rivalry than brother-brother dyad) and temporally within a sibling dyad (e.g., more during- than post-season). Furthermore, Davis and Meyer (2008) highlighted that older siblings might be motivated to maintain superior athletic status in the family, while younger siblings might be motivated to move out of the shadow of their sibling. Their findings also suggested a greater rivalry existed between siblings who were born closer together. In contrast, Blazo et al. (2014) and Côté (1999) both suggested such a closeness in age might result in bitterness, jealousy, or envy. More specifically, Côté highlighted that this took place during the investment years of development (16+ years; Côté, 1999). Crucially, such a relationship, and the emotional potency it can conjure, has the potential to result in dropout or burnout, as well as negatively impact on the wider family dynamic if not managed effectively (Fraser-Thomas et al., 2008).

Of course, there is a need to recognize that the influence, or even the very existance, of such abritary stages of development seems to be a psychosocial phenomenon. For example, Bridge and Toms (2013) found that the stages suggested by Côté were not as prevalent in a large sample of UK-based athletes, with specific socio-political influences (such as educational transitions) modifying the sampling, specializing, and investment stages within the Developmental Model of Sports Participation (DMSP: Côté, 1999). In short, coaches and psychologists must carefully consider the psychosocial milieu within which they are operating, especially when trying to import guidlines developed in other national setups.

In summary, the use and impact of rivalry between siblings on TD requires an “it depends” approach, primarily toward; birth order, gender, positive, and negative interpretations by each sibling, and the social milieu within which development has taken place. In short, siblings can matter, but exactly how is less straightforward.

## Supporting Practitioners: Building a Toolbox to Navigate the Complexity

Having highlighted the unique, broad, and complex contribution the sibling relationship can play in TD, it is important that practitioners are supported with tools that can help them to understand and utilize the relationship. As such, effective decision making by considering the TDE context and available options for action must be paramount. A good starting point for practitioners is to engage in critical reflection, asking; when should this be used (and when not)? with whom (and whom not)? where (and where not)? and crucially, why (and why not) (Cruickshank and Collins, 2016)? To illustrate, assuming that all siblings are competitive, and therefore should always play against each other, will not enhance development opportunities for all siblings in TD all of the time. For example, a large age-gap between same-sex siblings may lead to constant failure for one. Indeed, research demonstrates that excessive or ill-targeted sibling rivalry can be a major source of subsequent challenge to mental health (Tucker and Finkelhor, 2017).

Of course, there are genuine advantages to be gained, as can be seen from established research within more objectified environments, such as academic development. Described as “sibling spillover,” Nicoletti and Rabe (2014) suggest that small but significant positive impacts on academic attainment can cascade from older to younger siblings. Importantly, however, these are seen as related to specific behaviors, including older siblings helping the younger with homework or acting as an effective role model for positive behaviors. The point here is clear. Positive benefits can accrue if appropriate behavioral relationships exist. Whilst the extent to which coaches or psychologists (or perhaps even parents) can influence this is unclear, one key message is that such benefits require active encouragement and facilitation rather than being left to emerge spontaneously (Collins and MacNamara, 2018).

Expanding upon this mechanistic approach (i.e., exactly how and on what may sibling influence be positive), is the role of older siblings as agents of socialization (Kramer and Conger, 2009; Kramer, 2010). In simple terms, whilst first born children tend to be parent-focused in learning about the appropriateness of behaviors, those who follow tend to acquire more from their older siblings. As one of several results, younger siblings may acquire better insights into peer interactions, simply because their role model (their older brother or sister) is closer to the environment (Kramer and Conger, 2009). This can be particularly useful when the older sibling is in the same, or a parallel, sporting environment, as shown perhaps by the positive sibling examples highlighted earlier.

In order to support practitioners in navigating the complexity of this relationship by adopting an expertise approach, several hypothetical evidence-based examples are provided of sibling relationships to acknowledge both supportive and disruptive relations (see Table 2). Finally, the following section unpacks such considerations from the perspective of a sport psychologist and a coach, by providing implications for how to identify the characteristics of an individual relationship and possible options for action.

**Table 2:** Sibling relationship examples.

**Type of relationship**	**Biopsychosocial characteristics**	**Components**	**Context**	**Opportunities to accentuate**	**Opportunities to counter**
**Harmonious**	Interactional Context Emotional Interpersonal Skills	*Practice* *Play* *Closeness* *Support* *Empathy*	**Same sport**	Encourage technical skill development through practice in informal environments, with support, mentoring, co-operation and feedback and/or discussion	Encourage points of separation (time/distance) as athletes develop to allow individualized interpretation of sporting experiences with coaches and/or peers
	Skill Development Communication Separation	*Mentoring* *Co-operation* *Discussion* *Feedback* *Sport-specific*	**Different sport**	Older sibling can provide mentor support through a desire to watch their sibling compete, providing both support and feedback, that can be discussed later and worked on through play or practice	To develop skills such as realistic performance evaluation siblings may provide a good source of criticism at the right times, using their empathetic relationship to deliver this in a constructive manner
**Non-Harmonious**	Interactional Context Rivalry Skill Development Conflict	*Competition* *Motivation* *Success* *Challenge* *Arguments* *Frustration*	**Same sport**	Use the desire for sibling to create their own identity as an opportunity to grow self-regulatory skills on an individual basis. Potential to tap into level of rivalry to support and grow these skills	If rivalry becomes too intense it may lead to burnout or dropout. As such, trying to develop skills such as empathy through an external perspective (i.e., the coach or parent)
	Resilience Identity Separation	*Criticism* *Test* *Self* *Self-initiated*	**Different sport**	Encourage physical challenges to take place using motivation to win as a resource for developing key skills such as focus and coping with pressure	Encouraging siblings to spend more time together discussing how their specific skillset might benefit the other in their own sport

## So how Might you do it? Considerations for Coaches and Sport Psychologists

As highlighted by Cruickshank and Collins (2016), in order to make optimal decisions about the potential role of siblings in their TDE, it is important that practitioners take time to consider, and reflect on, a number of factors through the lens of their specific context. Firstly, the age and stage of an athlete's development will impact on the type of action a coach may take. For example, when the athlete concerned is 12-years old and the relationship is characterized by high levels of play (Interactional Context) and co-operation (Skill Development), siblings may be encouraged to create and play games that develop broader movement skills. From a biopsychosocial perspective, this might help an athlete develop muscular endurance (biological), planning, and evaluation (psychological), and communication and collaboration (social) skills. In comparison, a 16-year old athlete may be encouraged to practice (Interactional Context) a specific movement skill within the sport they participate in and seek feedback (Communication) from their sibling. Thus, developing the athlete biologically (e.g., agility), psychologically (e.g., focus), and socially (e.g., understanding) all within the same decision.

**Figure 1:**
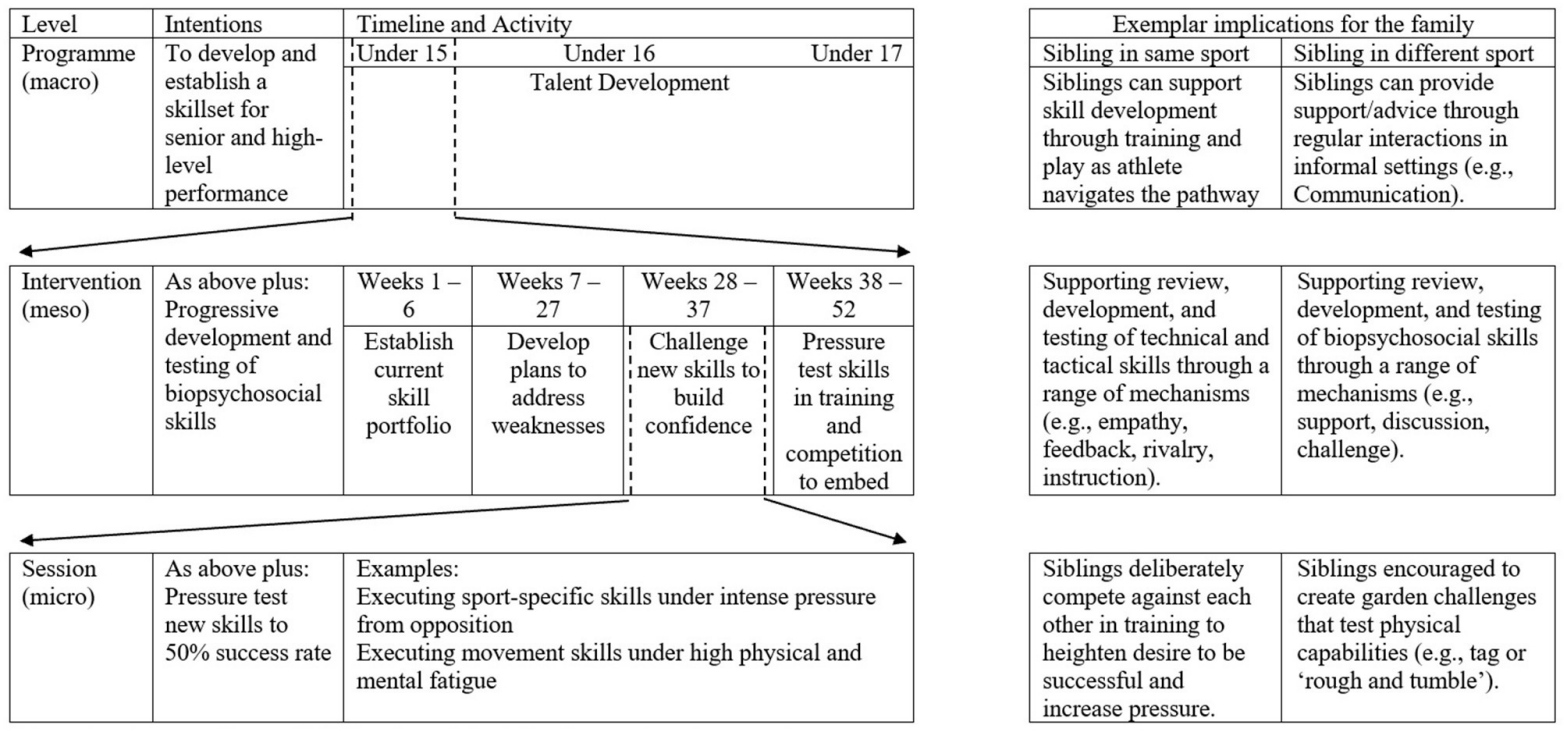
An exemplar nested plan for a talent development environment.

Furthermore, practitioners may consider how they can utilize the sibling relationship at different levels of planning. Consider Figure 1, a nested plan focusing on TD. Grounded in the coaching literature that underpins the Professional Judgement and Decision Making (PJDM) of practitioners, nested planning encourages coaches to engage in thinking at multiple levels of practice (Martindale and Collins, 2012). With greater coherence, this serves to maximize the potential to fulfill their intention for impact (Abraham and Collins, 2011; Martindale and Collins, 2012). Such an approach also facilitates the practitioner to frame the situation and conceptualize the issues involved (Martindale and Collins, 2012). Practitioners should consider where short-term goals (session) should be nested within medium (intervention) and then long-term (program) goals. In essence, a single intervention, should always have a purpose toward the longer-term objectives of the environment (Abraham et al., 2014). Indeed, such an approach fosters an improved understanding of important contextual demands, and therefore promote more efficient and accurate decisions for action that are aligned to the aims and objectives of any given phase of the TDE (Abraham and Collins, 2011). For example, consideration at a program level may see siblings utilized pre-season to increase motivation for the upcoming season. Following, they may be included at an intervention phase to support athletes interpret and respond to challenging experiences. Whilst at a session level, siblings playing the same sport may be used to create such challenge, by competing against each other. In short, a nested plan can provide a useful framework for planning and implementing effective strategies to engage sibling development (Abraham et al., 2014). Importantly though, as Collins L. et al. (2016) suggest, operationalizing decision making through biases based on generalizable competencies (e.g., all siblings are competitive), as opposed to more complex metacognitive skills (e.g., I have identified that sibling set A are highly competitive, but sibling set B are co-operative), will not allow us to optimally understand, explain, and support effective TD.

In summary, but also in keeping with good practice for all athletes, case conceptualization and subsequent PJDM (Martindale and Collins, 2005, 2007) are absolutely essential considerations when working with siblings. As such, this paper has highlighted many considerations which need to be addressed in developing an optimum plan for each individual. Of course, more research is needed to further understand such a complex relationship and its interaction with the talent environment. For example, a deeper exploration into individual mechanisms identified across the literature, and coaches' perceptions while coaching siblings or twins would represent a potentially beneficial next step. As our final point, and the essential take home from this paper, we refer to the website for educators, “we are teachers.” In 2018, they offered five rules for effective teaching of siblings. Notably, whilst the first four were two pairs of complete contradictions, the final was “There are absolutely *zero* rules when it comes to teaching siblings. So just sit back and enjoy the ride” (WeAreTeachers, 2018). We hope coaches and psychologists will do so too!

## Author Contributions

RT applied findings from his PhD thesis to the manuscript, writing the first manuscript draft. DC applied his expertise in psychology across the manuscript with a focus on the application to talent development. HC developed the final manuscript draft and reviewed and edited the final manuscript. All authors contributed to the manuscript revision and approved the definitive manuscript.

## Conflict of Interest

DC is a private practitioner operating through Grey Matters Performance Ltd. The remaining authors declare that the research was conducted in the absence of any commercial or financial relationships that could be construed as a potential conflict of interest.
